# High-Pressure Behavior of Ca_2_SnO_4_, Sr_2_SnO_4_, and Zn_2_SnO_4_

**DOI:** 10.1021/acs.jpcc.3c06726

**Published:** 2024-01-12

**Authors:** Simone Anzellini, Daniel Diaz-Anichtchenko, Josu Sanchez-Martin, Robin Turnbull, Silvana Radescu, Andres Mujica, Alfonso Muñoz, Sergio Ferrari, Laura Pampillo, Vitaliy Bilovol, Catalin Popescu, Daniel Errandonea

**Affiliations:** † Departamento de Física Aplicada-ICMUV, MALTA Consolider Team, Universidad de Valencia, Edificio de Investigación, Carrer del Dr. Moliner 50, 46100 Burjassot, Valencia, Spain; ‡ Departamento de Física, MALTA-Consolider Team, Instituto de Materiales y Nanotecnología, Universidad de La Laguna, San Cristóbal de La Laguna, E-38200 Tenerife, Spain; § Facultad de Ingeniería, Departamento de Física, Laboratorio de Sólidos Amorfos, Universidad de Buenos Aires, Av. Paseo Colón 850, C1063ACV Buenos Aires, Argentina; ∥ CONICET—Universidad de Buenos Aires, Instituto de Tecnologías y Ciencias de la Ingeniería “Hilario Fernández Long” (INTECIN), Av. Paseo Colón 850, C1063ACV Buenos Aires, Argentina; ⊥ Academic Centre for Materials and Nanotechnology, AGH University of Science and Technology, Al. Mickiewicza 30, 30-059 Krakow, Poland; # CELLS-ALBA Synchrotron Light Facility, Cerdanyola 08290, Barcelona, Spain

## Abstract

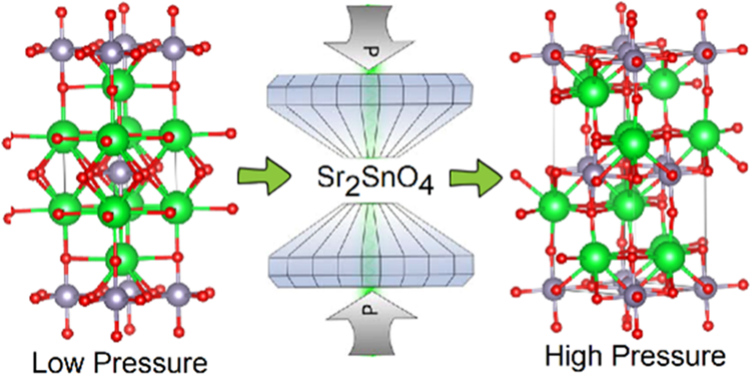

The pressure-induced structural evolution of Ca_2_SnO_4_, Sr_2_SnO_4_, and Zn_2_SnO_4_ has been characterized by powder X-ray diffraction up to 20 GPa using the ALBA synchrotron radiation source and density functional theory calculations. No phase transition was observed in Ca_2_SnO_4_ and Zn_2_SnO_4_ in the investigated pressure range. The observation in Zn_2_SnO_4_ solves contradictions existing in the literature. In contrast, a phase transition was observed in Sr_2_SnO_4_ at a pressure of 9.09 GPa. The transition was characterized as from the ambient-condition tetragonal polymorph (space group *I*4/*mmm*) to the low-temperature tetragonal polymorph (space group *P*4_2_/*ncm*). The linear compressibility of crystallographic axes and room-temperature pressure–volume equation of state are reported for the three compounds studied. Calculated elastic constants and moduli are also reported as well as a systematic discussion of the high-pressure behavior and bulk modulus of M_2_SnO_4_ stannates.

## 1 Introduction

**Figure 1:**
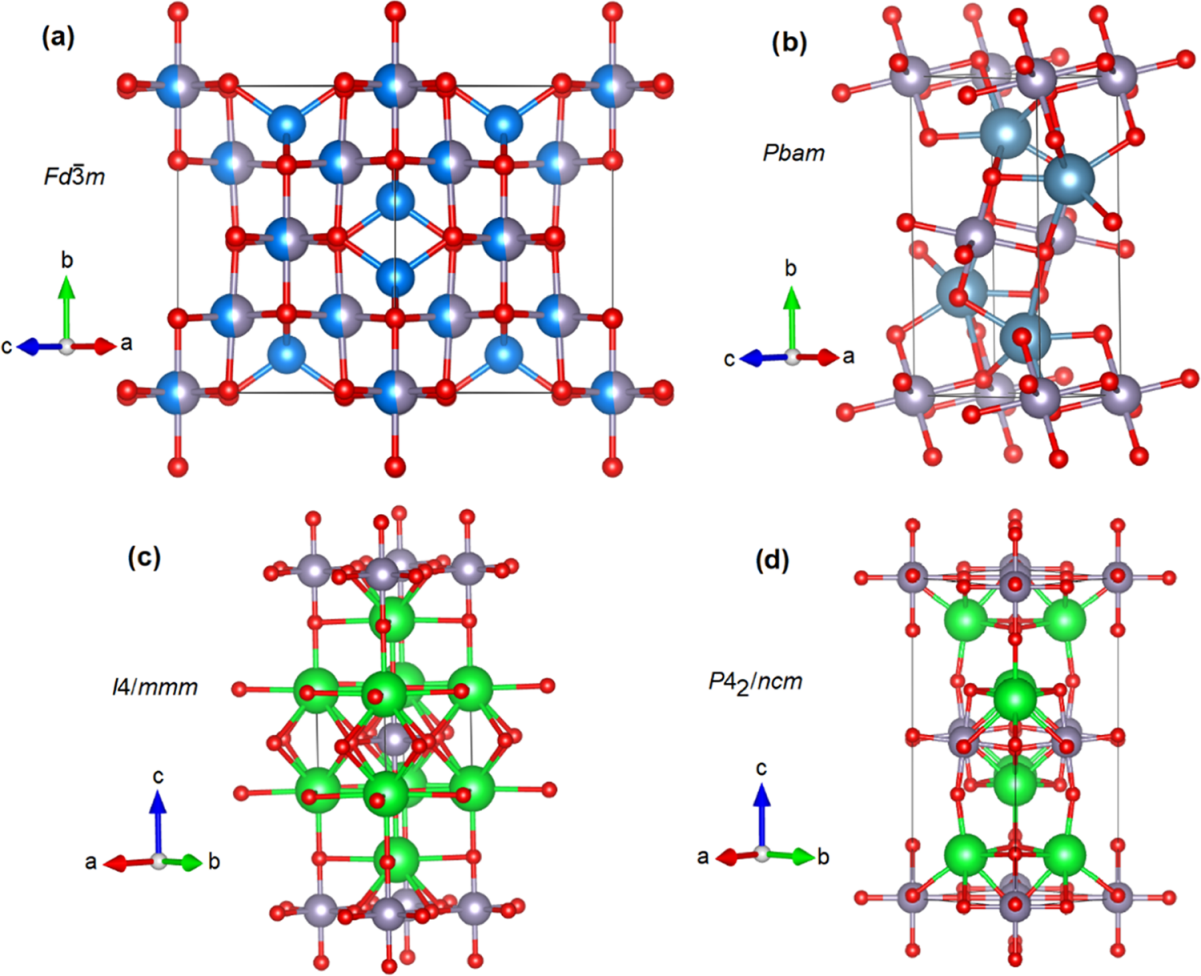
(a) Crystal structure of Zn_2_SnO_4_; Zn atoms are in blue, oxygen atoms in red, and blue/gray spheres represent the atomic positions occupied 50% by zinc and 50% by tin. (b) Crystal structure of Ca_2_SnO_4_; Ca atoms are in light blue, oxygen atoms in red, and tin atoms in gray. (c) Crystal structure of the tetragonal structure of Sr_2_SnO_4_ described by space group *I*4/*mmm*; Sr atoms are in green, oxygen atoms in red, and tin atoms in gray. (d) Crystal structure of the tetragonal structure of Sr_2_SnO_4_ described by space group *P*4_2_/*ncm*; Sr atoms are in green, oxygen atoms in red, and tin atoms in gray. Space groups of each structure are indicated in the figure.

M_2_SnO_4_ stannates, where M^2+^ = Mg, Mn, Ca, Ba, Sr, Pb, Cd, or Zn, are a fascinating family of compounds. They have been extensively studied due to their wide range of applications, including their use in photocatalysis,^1^ as electrode materials in Li-ion batteries,^2^ as anode materials in solar cells,^3^ and as phosphors when doped with lanthanide elements.^4^ A common characteristic of these stannates is that the Sn atoms in their crystal structure are six-coordinated, forming SnO_6_ octahedral units. M_2_SnO_4_ stannates are generally found in three typical structures. The first of these characteristic structures is the inverse cubic spinel structure (space group *Fd*3̅*m*),^5^ shown in Figure 1a, which has been observed for Cd_2_SnO_4_, Mg_2_SnO_4_, Mn_2_SnO4, and Zn_2_SnO_4_. In this structure, all M cations and half of the Sn cations occupy octahedral sites, while the other half of the Sn cations occupy tetrahedral sites. The second typical structure is orthorhombic^6^ and has been observed for Ca_2_SnO_4_, Cd_2_SnO_4_, and Pb_2_SnO_4_. The structure is described by space group *Pbam* and is isomorphic to Pb_2_PtO_4_. It is shown in Figure 1b. In this structure, the SnO_6_ octahedra form chains by sharing edges. The third typical structure is a first-series Ruddlesden–Popper structure which is isomorphic to K_2_NiF_4_, and it is described by the space group *I*4/*mmm.*^7^ This structure has been observed for Ba_2_SnO_4_ and Sr_2_SnO_4_. It is shown in Figure 1c. It has tetragonal symmetry and consists of alternating rock salt (MO) and perovskite layers (SnO_3_). In this structure, the SnO_6_ octahedra form chains of octahedral units by sharing corners in a plane. Two other polymorphs, which are very similar to each other, have also been observed in Sr_2_SnO_4_ upon cooling.^8,9^ One is orthorhombic and isomorphic to CuLa_2_O_4_.^8^ This structure is described by the space group *Pccn*. The other low-temperature polymorph is tetragonal and isomorphic to La_2_NiO_4_ and is described by the space group *P*4_2_/*ncm*. This structure can also be obtained under high-pressure (HP) conditions at room temperature as we will show in this work. The structure is shown in Figure 1d. This structure can be derived from the tetragonal K_2_NiF_4_ structure by tilting the SnO_6_ octahedra along the tetragonal *a*- and *b*-axes with nonequal tilts.

In addition to their technological relevance, M_2_SnO_4_ stannates have also attracted attention from fundamental research. Studies have been focused on the characterization of their temperature-induced phase transitions.^9−12^ In contrast, few studies have focused on the characterization of the pressure-induced behavior. In particular, only two members of this group of stannates have been studied under the HP conditions. One of them is Zn_2_SnO_4_. This compound has been first studied using density functional theory (DFT) simulations.^13^ By comparing the enthalpy as a function of pressure of the spinel, inverse spinel, and three common postspinel structures, it was proposed that subsequent phase transitions to titanite-type and ferrite-type structures could occur at 39 and 54 GPa, respectively.^13^ This result agrees with Raman and X-ray diffraction (XRD) experiments, which detected a phase transition in Zn_2_SnO_4_ at 40 GPa.^14^ However, the two previously cited studies^13,14^ are in contradiction with Raman experiments reporting the onset of the transition at 12.9 GPa^15^ and with XRD studies which found gradual structural changes from 12.5 to 29.8 GPa, where a cubic–hexagonal transition was found.^16^ In addition, there are large discrepancies in the literature regarding the value of the bulk modulus of inverse-spinel-type Zn_2_SnO_4_.^14,15^ Values of 169^15^ and 242 GPa have been reported from experiments for this parameter, and a value of 189 GPa was obtained from DFT calculations.^13^ The above-described discrepancies could be related to the use of samples with different characteristics (for instance, single crystals or nanoparticles)^17^ or the use of different pressure media in the experiments.^18^ However, none of these facts can be used to explain the contradictions found in the literature. Regarding the pressure medium, in the three experiments,^14−16^ silicone oil was used as the pressure medium. Regarding the sample characteristics, in refs (14) and (15), similar nanoparticles were studied, but contradictory results were reported, and refs (15) and (16) reported a similar transition pressure in spite that in one case, nanoparticles were studied^15^ and in the other, the studied sample was a single crystal.^16^ The contradiction between previous works clearly supports the need for additional studies to confirm or rule out the existence of a phase transition in Zn_2_SnO_4_ around 12.5 GPa and to accurately determine the pressure dependence of the volume of Zn_2_SnO_4_. The second stannate which has been studied under HP is Pb_2_SnO_4_.^6^ In this stannate, structural phase transition occurs between 10 and 12 GPa, with a change of space group from *Pbam* to *Pnam*. The phase transition is related to the fact that the lone electron pairs of the Pb^2+^ ions form bonds on compression.^6^ The results summarized above suggest that it is timely to perform additional studies on the HP behavior of M_2_SnO_4_ stannates. In particular, studies focus on solving contradictions in results previously reported for Zn_2_SnO_4_ and in extending studies to compounds different than Zn_2_SnO_4_ and Pb_2_SnO_4_ to check their structural stability up to 20 GPa.

The objective of this study is to characterize the pressure-induced structural evolution of three different stannates. In this work, we present HP synchrotron powder XRD studies on Zn_2_SnO_4_, Ca_2_SnO_4_, and Sr_2_SnO_4_. We choose these three compounds as representatives of their respective characteristic structures: inverse cubic spinel (Zn_2_SnO_4_); Pb_2_PtO_4_-type (Ca_2_SnO_4_); and K_2_NiF_4_-type (Sr_2_SnO_4_). Such a characterization will contribute to a more systematic understanding of the HP behavior of M_2_SnO_4_ stannates up to 20 GPa. Furthermore, the study on Zn_2_SnO_4_ sheds light on the controversies observed between previous different studies.^13−16^ The study on Ca_2_SnO_4_ allows a direct comparison with the previous study on isostructural Pb_2_SnO_4_.^6^ The study on Sr_2_SnO_4_ is the first HP study on a tetragonal K_2_NiF_4_-type stannate. We found that Zn_2_SnO_4_ and Ca_2_SnO_4_ remain stable in their ambient-pressure structures up to 20 GPa. In contrast, Sr_2_SnO_4_ undergoes a phase transition at 9 GPa from the structure described by the space group *I*4/*mmm* to that described by the space group *P*4_2_/*ncm*. The phase transition is reversible. The compressibility of the three studied compounds is also reported including an empirical relationship that can be used to estimate the bulk modulus of unstudied stannates.

## 2 Methods

Polycrystalline Ca_2_SnO_4_ was synthesized by a co-precipitation method. Stoichiometric amounts of CaCl_2_·2H_2_O and Na_2_SnO_3_ (2 Ca: 1 Sn) were diluted in distilled water. A solution of sodium oxalate Na_2_C_2_O_4_ was added dropwise to the obtained mixture. The white precipitate was washed until the pH reached 7 and dried for 24 h at 100 °C. We confirmed by powder XRD that the synthesized sample was Ca_2_SnO_4_, with an orthorhombic structure described by space group *Pbam* and with unit-cell parameters *a* = 5.7496(4) Å, *b* = 9.6990(7) Å, and *c* = 3.2658(3) Å, which agree with the literature.^19^ Polycrystalline Sr_2_SnO_4_ was synthesized by the same co-precipitation method with the only difference that we started the synthesis from stoichiometric amounts of Sr(NH_3_)_2_ and Na_2_SnO_3_ (2 Sr: 1 Sn) diluted in distilled water. In this case, XRD confirmed the synthesis of the tetragonal structure described by space group *I*4/*mmm* with unit-cell parameters *a* = 4.0532(3) Å and *c* = 12.600(2) Å, which agree with the literature.^7^ We also detected a small amount of SnO_2_. The co-precipitation method used to synthesize Zn_2_SnO_4_ was different. For this compound, stoichiometric amounts of ZnCl_2_ and SnCl_4_·5H_2_0 (2 Zn: 1 Sn) were diluted in distilled water to reach a 0.1 molar concentration. A 0.5 molar solution of NaOH was added dropwise to the starting solution. The white precipitate was washed until the pH reached 7 and then dried for 24 h at 100 °C. The dried product was heated at 1000 °C for 7 h. We obtained inverse cubic spinel-type Zn_2_SnO_4_ (space group *Fd*3̅*m*) and a small fraction of impurities of wurtzite-type ZnO. The unit-cell parameter of Zn_2_SnO_4_ was *a* = 8.6828(2) Å, which agrees with the literature.^5^

In the HP powder XRD measurements, we used fine powders obtained by grinding the synthesized polycrystalline samples. The experiments were performed at the ALBA-CELLS synchrotron using the BL04-MSPD beamline.^20^ We used different membrane-type diamond-anvil cells (DACs). We used diamonds with 500 μm diameter culets and a preindented stainless-steel gasket with a central hole 200 μm in diameter. The high-pressure chambers of the DACs were loaded with the sample and some copper (Cu) powder. A 4:1 methanol–ethanol mixture was used as a pressure-transmitting medium. This pressure medium provides quasi-hydrostatic conditions up to 10 GPa,^21^ but it is normally used to obtain high-quality results from oxides up to 30 GPa.^22^ The pressure medium was selected to provide similar experimental conditions than in refs (14,15)–^16^. The pressure inside the cell was obtained from the volume of Cu extracted from the XRD signal, following the equation of state of Dewaele et al.^23^ The wavelength of the monochromatic X-ray beam was 0.4642 Å, and the spot size was 20 μm × 20 μm (full width at half-maximum). We used a Rayonix SX165 CCD instrument to collect the diffraction patterns. Masks were applied on a per-image basis, and the images were azimuthally integrated using the DIOPTAS suite.^24^ A Pawley refinement of the obtained data was then performed using the TOPAS suite^25^ using literature values as starting parameters.

Total energy calculations at zero temperature were performed within the ab initio framework of the density functional theory (DFT),^26^ as implemented in the Vienna Ab initio Simulation Package (VASP),^27^ using the projector augmented-wave scheme (PAW).^28,29^ The generalized-gradient approximation (GGA) to the exchange-correlation energy used in the calculations was that of Perdew, Burke, and Ernzerhof for solids, or PBEsol.^30^ The cutoff in the kinetic energy of the plane-wave basis set used in the calculations was 560 eV, which ensured highly converged results. Brillouin zone (BZ) integrations were performed using 6 × 6 × 8, 4 × 2 × 8, and 6 × 6 × 6 Monkhorst–Pack integration grids, respectively, for the structures considered for Sr_2_SnO_4_, Ca_2_SnO_4_, and Zn_2_SnO_4_.

In order to obtain the equilibrium configuration at each fixed volume corresponding to hydrostatic pressure conditions, the unit-cell parameters and the atomic positions were fully relaxed to a tolerance of the residual forces and deviations of the stress tensor from a diagonal hydrostatic form of less than 0.003 eV/A and 0.1 GPa, respectively. The set of energies and volumes/pressures provided by the simulations were fitted with a Birch–Murnaghan equation of state^31^ to obtain the zero-pressure equilibrium volume, bulk modulus, and its pressure derivative.

The mechanical properties were studied through the calculation of stress–strain relations (elastic constants) implemented in VASP using the Le Page methodology.^32^ From the calculated full set of elastic constants, various elastic moduli were readily obtained. Lattice dynamic calculations of the phonon modes were carried out at the zone center of the BZ (Γ point) using the direct force-constant approach (small displacements method).^33^ These calculations provide not only the frequency of the normal modes but also their polarization vectors and symmetry, which allows us to assign the irreducible representations and the character and optical activity of the modes at the Γ-point.

## 3 Results and Discussion

### 3.1 Zn_2_SnO_4_

**Figure 2:**
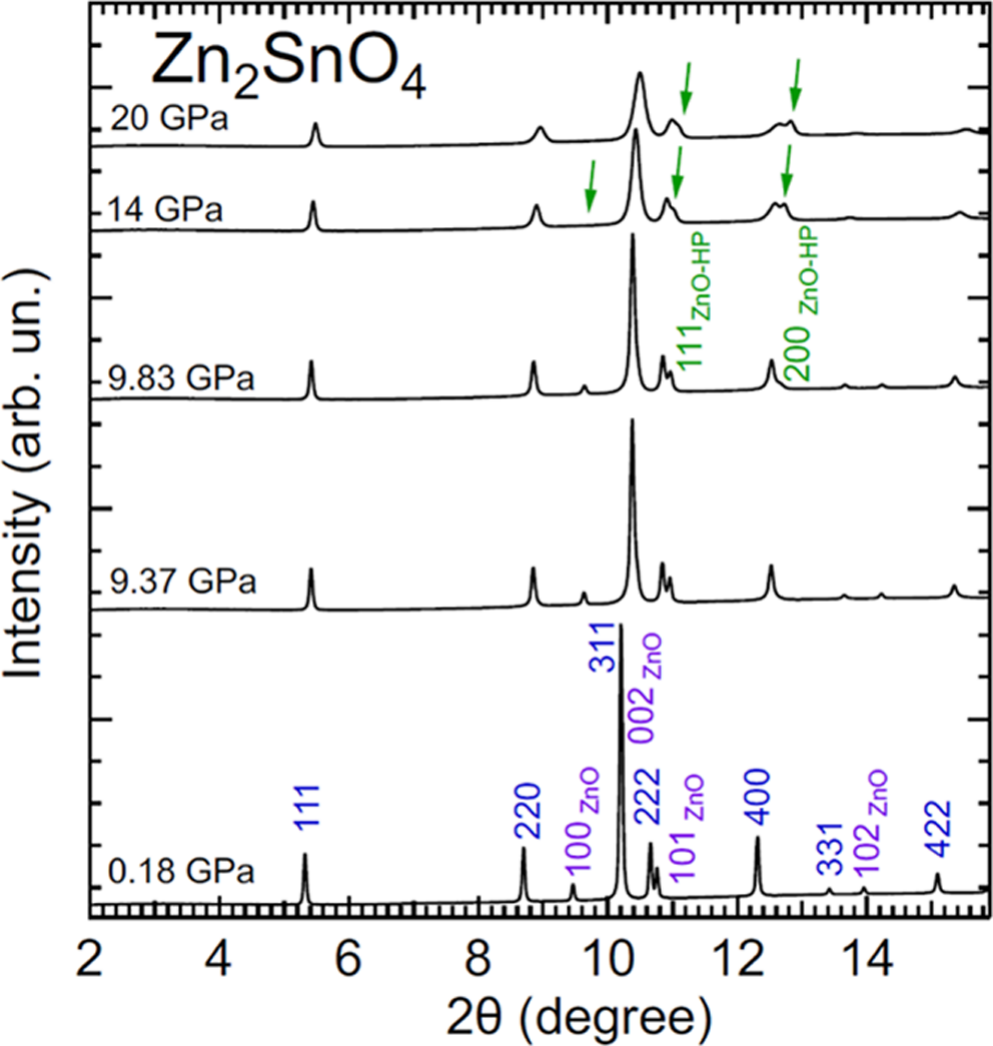
Selection of XRD patterns measured in Zn_2_SnO_4_ at different pressures (indicated in the figure) with an X-ray wavelength of 0.4642 Å. Peaks from Zn_2_SnO_4_ and ZnO are identified with blue and purple labels, respectively. Green labels and arrows are used for the HP phase of ZnO.

Figure 2 shows a selection of powder XRD patterns obtained for Zn_2_SnO_4_ up to 20 GPa (a typical Pawley fit to XRD patterns is shown in Figure S1 in the Supporting Information). In the bottom XRD pattern, we identify the peaks assigned to Zn_2_SnO_4_ using Miller indices. We also identify the peaks assigned to the ZnO impurity. The ZnO peaks do not overlap with those of Zn_2_SnO_4_, thereby not precluding the study of the pressure effects on the crystal structure of this compound. With increasing pressure, we observed the shift of the peaks toward higher angles due to the contraction of lattice parameters. At 9.83 GPa, we observed the appearance of extra peaks. The presence of these peaks is due to the well-known phase transition of ZnO.^34^ We did not find any evidence of the occurrence of a phase transition in Zn_2_SnO_4_, which remains in the cubic inverse-spinel structure up to 20 GPa, in agreement with the conclusions of DFT calculations^13^ and the experiments reported in ref (14). The structural changes and phase transitions reported in other studies^15,16^ could be related to the presence of large deviatoric stresses due to the bridging of the sample between diamonds.^35^ They cannot be related to the effect of using samples with nanometer particle sizes^36^ because both in refs (14) and (15), the studies were performed using nanoparticles. The influence of the pressure medium on results could be also excluded as the cause of the observation of phase transitions near 12.5 GPa in refs (15) and (16) and not in ref (14). The three previous experimental studies used the same pressure medium, silicone oil which is quasi-hydrostatic up to 4 GPa.^21^ In addition, our study performed under 4:1 methanol–ethanol, which is quasi-hydrostatic up to 10 GPa,^21^ supports the structural stability of the inverse-spinel structure as in ref (14), where silicone oil was the pressure medium.

**Figure 3:**
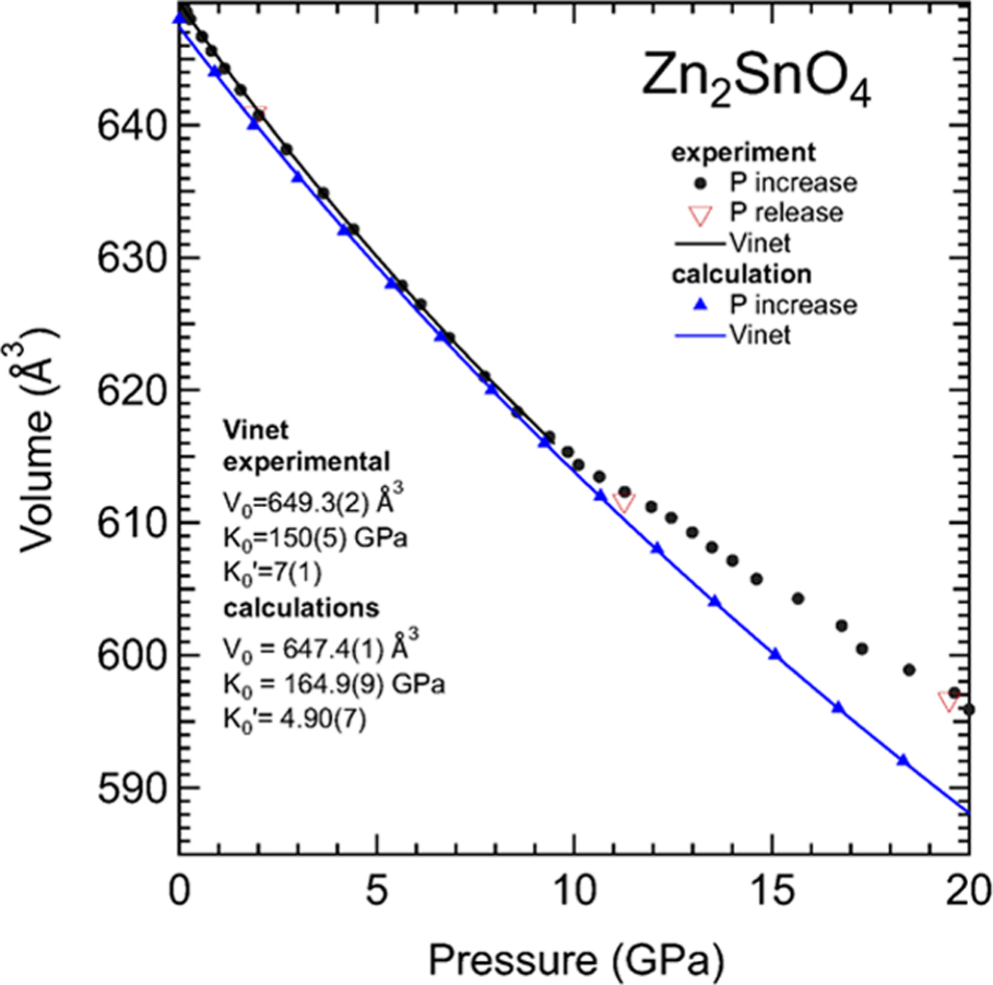
Pressure dependence of the unit-cell volume of Zn_2_SnO_4_. Black circles (red triangles) are results obtained during compression (decompression). Blue triangles are results from calculations. The black and blue lines represent the Vinet EOS described in the text for the experiment and calculations, respectively. Errors bars are smaller than symbols.

From the powder XRD patterns, we determined the pressure dependence of the unit-cell parameter *a* of the cubic structure. A table with these results is included in the Supporting Information (Table S1). In Figure 3, we plot the unit-cell volume versus pressure, including also the results from DFT calculations. The agreement between calculations and experiments is excellent up to 10 GPa, which coincides with the limit of quasi-hydrostaticity for the used pressure-transmiting medium.^21^ Above 10 GPa, there is a decrease in the compressibility in the experimental results. This is a typical phenomenon caused by nonhydrostatic stresses.^37^ From the experiments up to 10 GPa (quasi-hydrostatic regime), we have fitted the results shown in Figure 3 using a third-order Vinet equation of state (EOS).^38^ From the experimental data, we obtained the volume at zero pressure *V*_0_ = 649.3(2) Å^3^, the bulk modulus *K*_0_ = 150(5) GPa, and its pressure derivative *K*_0_′ = 7(1). If a second-order Vinet EOS is used assuming the same volume, the other two parameters are *K*_0_ = 160(5) GPa and *K*_0_′ = 4. There is good agreement with the results obtained from DFT calculations up to 20 GPa, *V*_0_ = 647.4(1) Å^3^, *K*_0_ = 164.9(9) GPa, and *K*_0_′ = 4.90(7). Our results also agree with those previously obtained from nanowires of Zn_2_SnO_4_ (*K*_0_ = 168.6(9.7) GPa and *K*_0_′ = 4).^15^ This agreement suggests that previous DFT calculations using the Crystal09 code and a Becke three-parameter hybrid nonlocal exchange functional combined with the Lee–Yang–Parr functional have slightly overestimated the bulk modulus (*K*_0_ = 168.6(9.7) GPa and *K*_0_′ = 4) and that previous XRD studies have largely overestimated it (*K*_0_ = 241.52(2) GPa and *K*_0_′ = 4). These experiments were carried out using silicone as pressure medium and show a compressibility change at 5 GPa, the limit of quasi-hydrostaticity for this medium.^21^ If data from this work for *P* < 5 GPa are refitted, a bulk modulus comparable to our study is obtained (*K*_0_ = 171(6) GPa and *K*_0_′ = 6(1)).

### 3.2 Ca_2_SnO_4_

**Figure 4:**
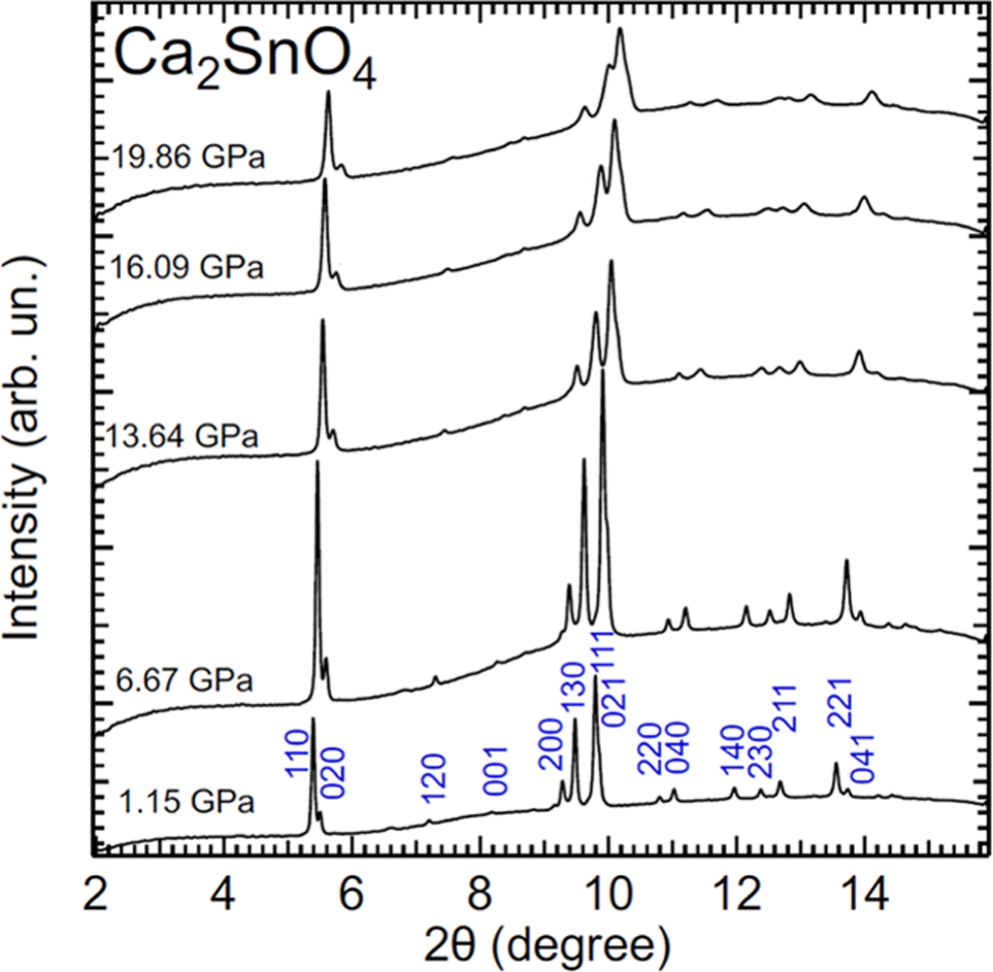
Selection of XRD patterns measured in Ca_2_SnO_4_ at different (indicated in the figure) wavelengths with an X-ray wavelength of 0.4642 Å. Peaks of the *Pbam* structure of the sample are identified with a Miller index at the lowest pressure.

Figure 4 shows powder XRD patterns measured in Ca_2_SnO_4_ at selected pressures of up to 19.86 GPa. In this experiment, in addition to peaks from the sample, there is a diffuse background from diamond anvils which does not affect the results of experiments. All peaks can be assigned to the orthorhombic structure of Ca_2_SnO_4_ from the lowest to the highest pressure (a typical Pawley fit to XRD patterns is shown in Figure S2 in the Supporting Information). In the bottom XRD pattern, we identify the peaks using Miller indices. As the pressure increases, the peaks shift toward higher angles due to the contraction of lattice parameters. At 13.64 GPa, we observe a broadening of the peaks, which is consistent with the development of nonhydrostatic stresses^39^ in the 4:1 methanol–ethanol mixture.^21^ According to our experiments, Ca_2_SnO_4_ remains in the orthorhombic structure up to the highest pressure covered by the experiments.

**Figure 5:**
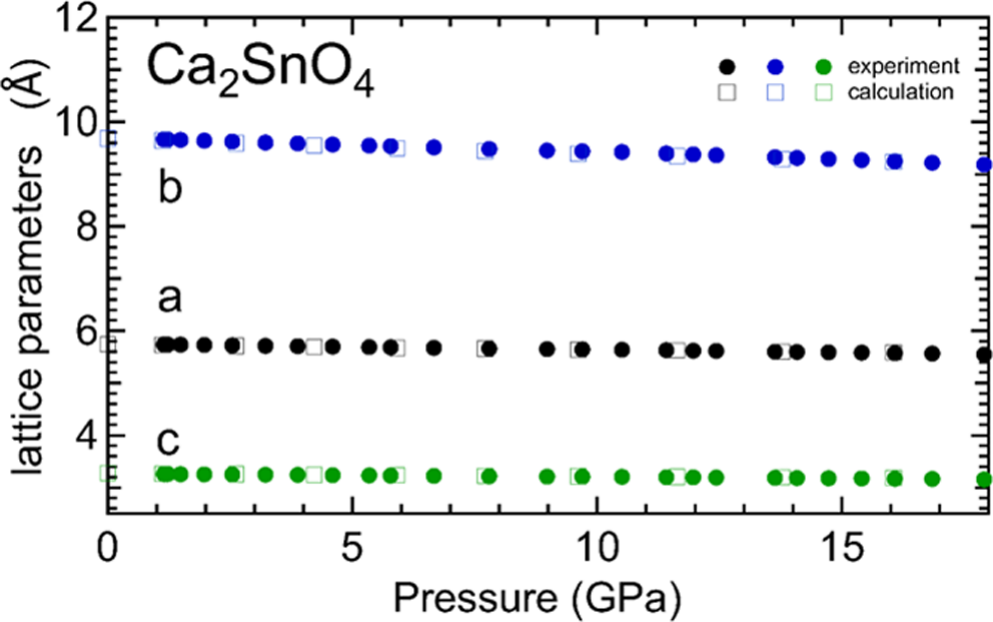
Pressure dependence of the unit-cell parameters of Ca_2_SnO_4_. Solid circles are from experiments, and empty squares are from DFT calculations. Errors bars are smaller than symbols.

**Figure 6:**
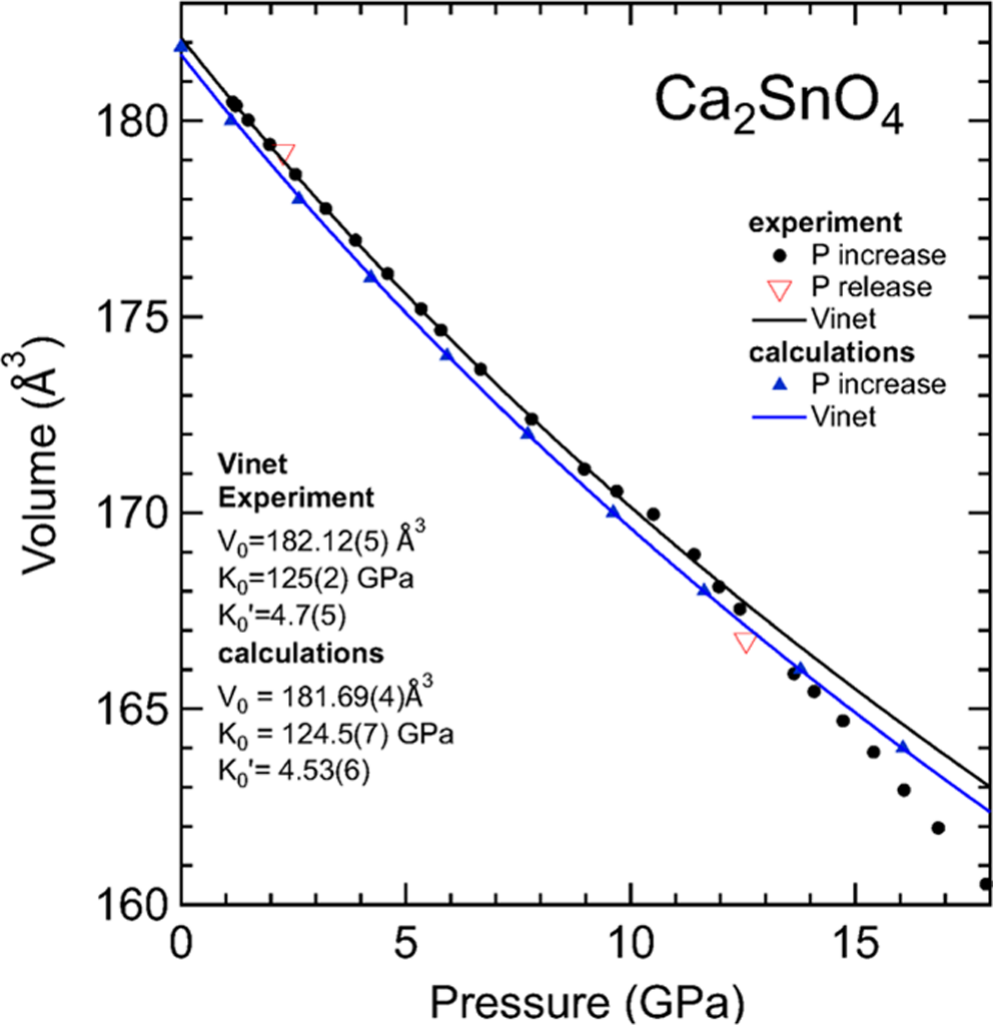
Pressure dependence of the unit-cell volume of Ca_2_SnO_4_. Black circles (red triangles) are results obtained during compression (decompression). Blue triangles are results from calculations. The black and blue lines represent the Vinet EOS described in the text for experiment and calculations, respectively. Errors bars are smaller than symbols.

In Figure 5, we show the pressure dependence of the unit-cell parameters we obtained from our experiments and calculations. A table with unit-cell parameters versus pressure obtained from experiments is included in the Supporting Information (Table S2). In Figure 6, we present the pressure dependence of the unit-cell volume. We found that the compressibility is anisotropic, with the *b*-axis being the most compressible axis (see Figure 5). The linear compressibilities of the axes are κ_a_ = 1.91(9) × 10^–3^ GPa^–1^, κ_b_ = 2.75(9) × 10^–3^ GPa^–1^, and κ_c_ = 1.87(9) × 10^–3^ GPa^–1^. The *b*-axis is 45% more compressible than the other two axes. The anisotropic compressibility can be explained by the fact that the crystal structure (shown in Figure 1b) has linear chains of edge-sharing SnO_6_ octahedra running along the *c*-axis which are connected along the *a*-axis by a Ca coordination polyhedron CaO_6–7_, which has been described in the literature as a 6- or 7-coordination polyhedron forming layers. They are separated from each other by CaO_6–7_ polyhedra layers. Since the Ca–O bonds (2.34–2.74 Å) are much larger than Sn–O bonds (2.00–2.14 Å), the CaO_6–7_ polyhedra are much more compressible than the SnO_6_ octahedra, which favors the compression in the direction perpendicular to the CaO_6–7_ polyhedra layers, i.e., along the *b*-axis.

In Figure 6, it can be seen that the agreement between calculations and experiments is excellent up to 12 GPa, the limit of quasi-hydrostaticity for the pressure-transmitting medium used.^21^ Above 13.64 GPa, the same pressure at which peak broadening is observed, the experimental data show a change in the compressibility of Ca_2_SnO_4_. As we argued in Zn_2_SnO_4_, we hypothesize that this is an artifact caused by nonhydrostatic stresses. However, the possibility of subtle phase transitions caused by the greater compressibility of the *b*-axis cannot be excluded. The answer to this question requires the performance of single-crystal XRD experiments.^40^ From the results obtained from experiments up to 10 GPa (quasi-hydrostatic conditions), we have fitted the pressure dependence of the volume using a third-order Vinet equation of state. From experiments, we obtained *V*_0_ = 181.12(5) Å^3^, *K*_0_ = 125(5) GPa, and *K*_0_′ = 4.7(5). There is excellent agreement with the results obtained from the DFT calculations up to 20 GPa, *V*_0_ = 181.69(4) Å^3^, *K*_0_ = 124.5(7) GPa, and *K*_0_′ = 4.53(6). These results show that Ca_2_SnO_4_ is more compressible than Zn_2_SnO_4_ (*K*_0_ = 150(5) – 164.9(9) GPa) and have a similar bulk modulus than the orthorhombic HP phase of Pb_2_SnO_4_ (*K*_0_ = 117 GPa).^6^ The systematic of the bulk modulus of M_2_SnO_4_ stannates will be discussed at the end of the manuscript when the elastic constants are also discussed.

### 3.3 Sr_2_SnO_4_

**Figure 7:**
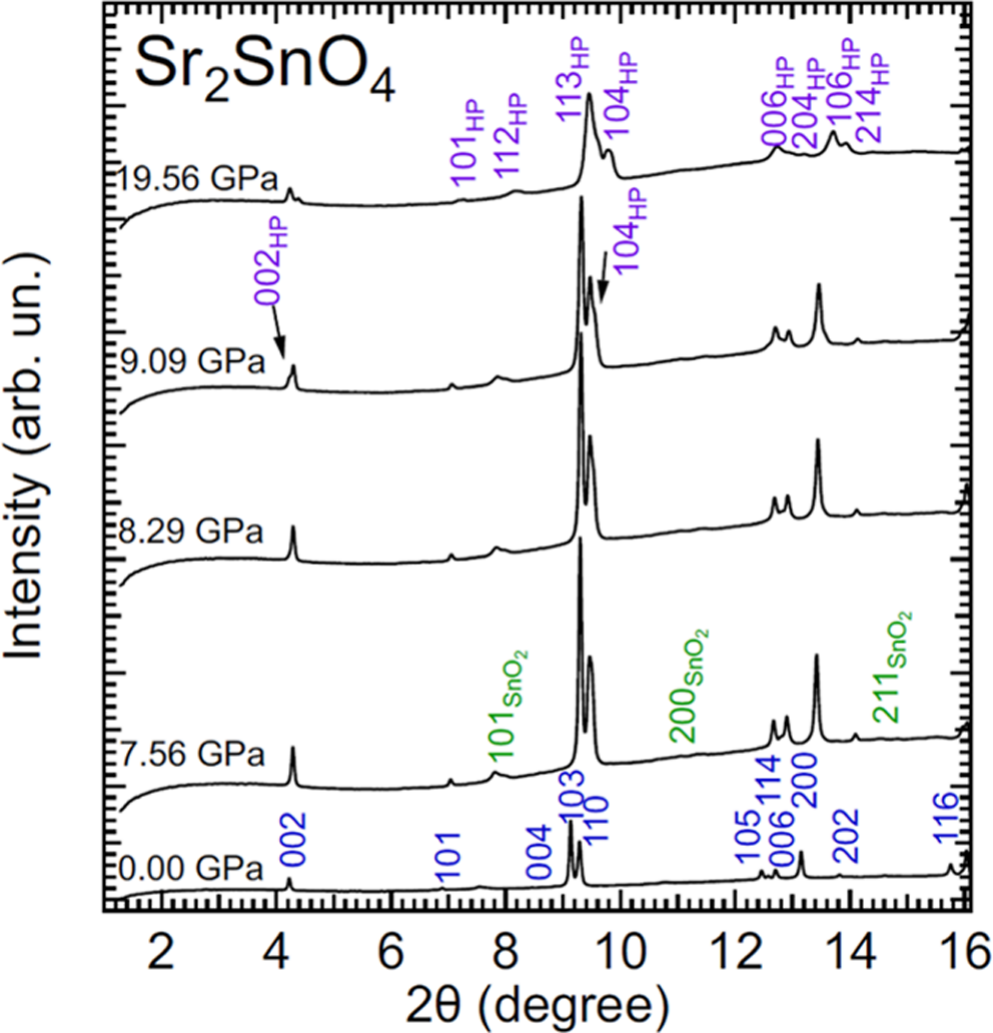
Selection of XRD patterns measured in Sr_2_SnO_4_ at different pressures (indicated in the figure) with an X-ray wavelength of 0.4642 Å. Peaks of the low-pressure (high-pressure) phase are identified with a Miller index in blue (violet). Miller indices in green identify weak peaks of SnO_2_.

Figure 7 shows a selection of XRD patterns measured in Sr_2_SnO_4_ up to 19.56 GPa (typical Pawley fits to XRD patterns are shown in Figures S3 and S4 in the Supporting Information). Up to 8.29 GPa, the XRD patterns can be assigned to the tetragonal phase of Sr_2_SnO_4_ (space group *I*4/*mmm*). The XRD patterns show the presence of extra weak peaks, which can be unambiguously assigned to SnO_2_. These peaks never overlap with those from the studied sample and, therefore, do not affect the phase identification and the determination of unit-cell parameters. At 9.09 GPa, additional peaks appear in the XRD pattern. The emergence of these peaks indicates the onset of a phase transition. The intensity of the additional peaks increases with increasing pressure, while the peaks of the low-pressure phase lose intensity. However, quantifying the changes in phase abundance from the intensity changes is not possible due to the influence of preferred orientations. The low- and high-pressure phases coexist up to the highest pressure covered by our experiments, 24.35 GPa. If the XRD patterns at 0 and 19.56 GPa are compared in Figure 7, it can be seen that around 4° there are two peaks at the highest pressure (due to phase coexistence) but only one peak at 0 GPa. The observed transition occurs at a pressure close to the nonhydrostatic limit of the 4:1 methanol–ethanol; therefore, there is a possibility that the observed transition could be an artifact caused by the fact that two grains could be under different effective pressures, and this could cause the observed peak splitting. For a definitive answer to this question, additional experiments performed using helium as a pressure-transmitting medium are needed. The phase transition is reversible with a small hysteresis (the low-pressure phase is recovered as a single phase at 5.3 GPa), and we do not find any evidence of a transition to the orthorhombic structure described by space group *Cmca* predicted by previous DFT calculations^41^ or to the orthorhombic phase observed at low temperatures (space group *Pccn*).^8^

We assigned the crystal structure of the HP phase to that of the tetragonal low-temperature polymorph (space group *P*4_2_/*ncm*). The unit-cell parameters of the HP phase at 9.09 GPa are *a* = 5.574(4) Å and *c* = 12.537(9) Å. Note that the *b* parameter is similar to that of the low-pressure phase and that the *a* parameter of the HP phase is nearly equal to the √2*a* of the low-pressure phase. This is because of the axis transformation between space groups which is given by the matrix . Consequently, there is a unit-cell doubling at the phase transition. The HP phase can be obtained from the low-pressure phase by the introduction of cooperative tilting of SnO_6_ octahedra.^9^ The transition under HP to a low-temperature polymorph is not unexpected. Such a phenomenon has been observed before in several oxides, e.g., SrMoO_4_,^42^ CaMoO_4_,^43^ and LaNbO_4_,^44^ after it had been postulated that there was an inverse relationship in oxides.^44^ Interestingly, there is no detectable volume change when comparing the unit-cell volume per formula unit of the low- and high-pressure phases at the same pressure. This and the relationship between the two space groups suggest that the transition might be second order in nature. However, in such a case, the two phases should not coexist (as observed here). There are two possible explanations for phase coexistence. One is that the transition is a weak first-order transition. The small jump in unit-cell parameters at the transition supports this hypothesis (see Figure 10). The second is that phase coexistence is caused by stresses between grains, which induces local deviatoric stresses, a fact recently noticed by comparing HP powder XRD experiments (showing phase coexistence) and HP single-crystal XRD experiments (not showing phase coexistence) in different oxides like FeVO_4_ and BiMnO_3_ at pressures as low as 3 GPa.^45,46^

**Figure 8:**
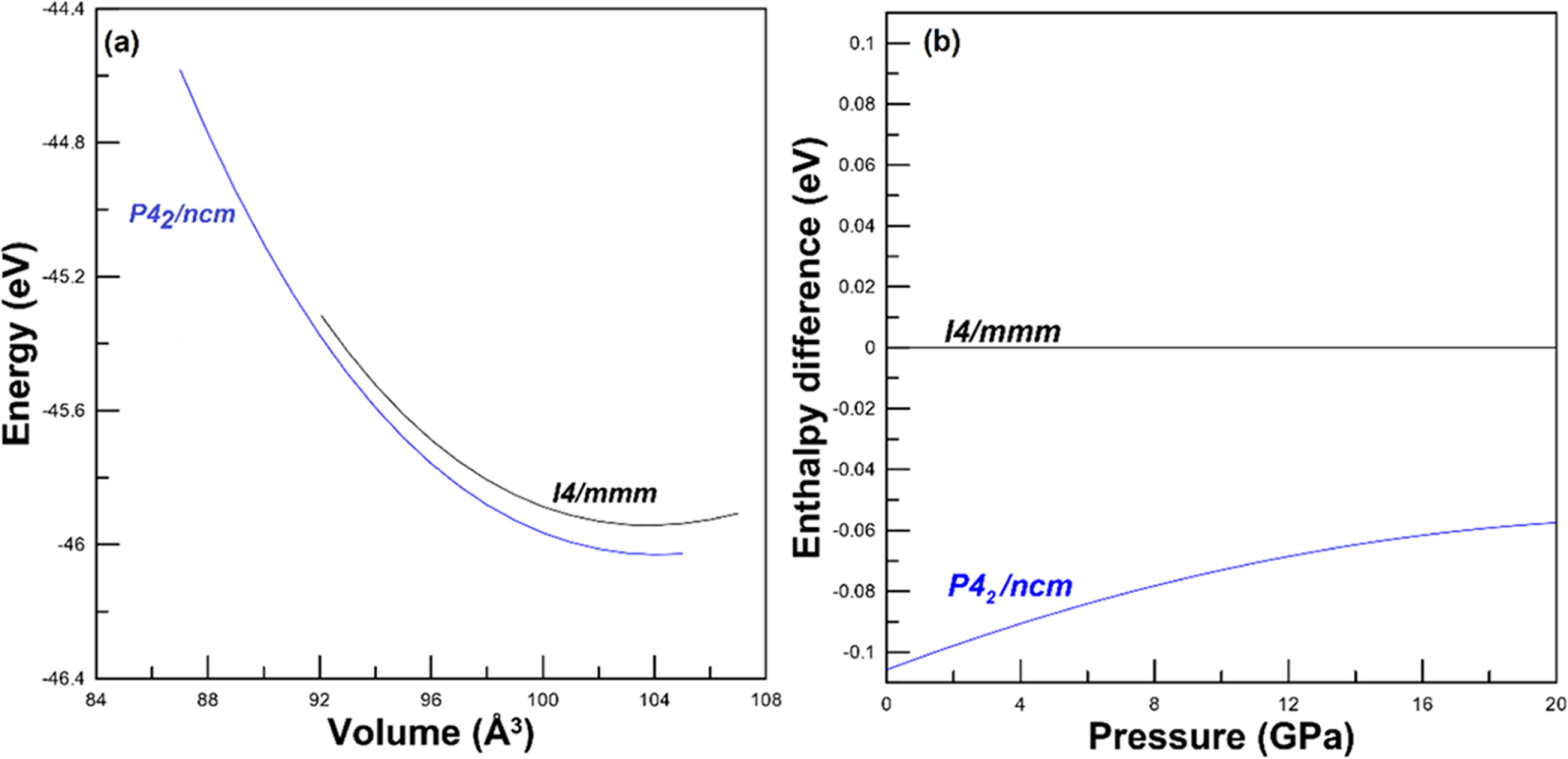
(a) Energy versus volume per formula unit for the two polymorphs of Sr_2_SnO_4_. (b) Enthalpy difference between the low- and high-pressure structures as a function of pressure. The low-pressure phase is used as a reference.

A possible explanation for the observed phase transition is the fact that the tetragonal polymorph has been found to be a metastable polymorph,^41^ with dynamical instabilities at ambient conditions which is observed due to the presence of a kinetic barrier. There are many examples of metastable oxide polymorphs reverting to the stable polymorph under a relatively low compression, as documented for example in BiPO_4._^47^ To support our hypothesis, in Figure 8a, we report calculations of the total energy of the two phases of Sr_2_SnO_4_, and in Figure 8b, we plot their enthalpies versus pressure. Figure 8a shows that the tetragonal HP (and low temperature) polymorph (P4_2_/*ncm*) is the lowest-energy polymorph. Figure 8b shows that within the pressures covered by our study, the HP tetragonal structure has a lower enthalpy than the ambient-pressure tetragonal polymorph (*I*4/*mmm*). Thus, the HP tetragonal structure is the most thermodynamically favored polymorph.

**Figure 9:**
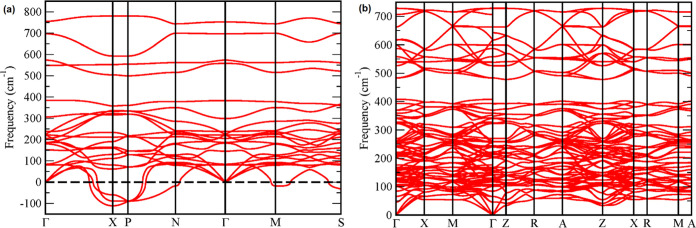
(a) Phonon dispersion of ambinet-pressure tetragonal Sr_2_SnO_4_ (*I*4/*mmm*). (b) Phonon dispersion of high-pressure tetragonal Sr_2_SnO_4_ (*P*4_2_/*ncm*).

To understand the causes of the phase transition, we have calculated the phonon dispersion of both structures, which are shown in Figure 9. In the figure, it can be seen that the structure described by space group *I*4/*mmm* has three negative branches which indicates that the structure is dynamically unstable. On the contrary, in the structure described by space group P4_2_/*ncm*, all phonon branches are positive, i.e., the structure is dynamically stable. Consequently, the ambient-pressure structure *I*4/*mmm* is a metastable structure. Consequently, pressure transforms the metastable structure (*I*4/*mmm*) into the stable structure, as observed in other oxides.^47^

We will compare now Sr_2_SnO_4_ with the other stannate undergoing a phase transition, Pb_2_SnO_4_.^6^ Interestingly, in Pb_2_SnO_4_, a phase transition has been observed at similar pressures to those in Sr_2_SnO_4_ (10–12 GPa).^6^ In the lead stannate, the transition was favored by the role played by lone electron pairs of Pb. This gives Pb_2_SnO_4_ a peculiar behavior under compression because the crystal structure becomes strongly distorted on compression with an elongation of one axis.^6^ In this compound, the transition involves the formation of bonds between Pb^2+^ ions. This is not the case for Sr_2_SnO_4_ where the phase transition causes only a slight distortion of the structure (compare Figure 1c,d) and no formation of new bonds. In fact, the transition involves only the tilting of SnO_6_ octahedra. Giving the similarities between the crystal structures of Sr_2_SnO_4_ and Ba_2_SnO_4_,^7^ we can speculate that the second compound would undergo a similar phase transition to that in Sr_2_SnO_4_.

**Figure 10:**
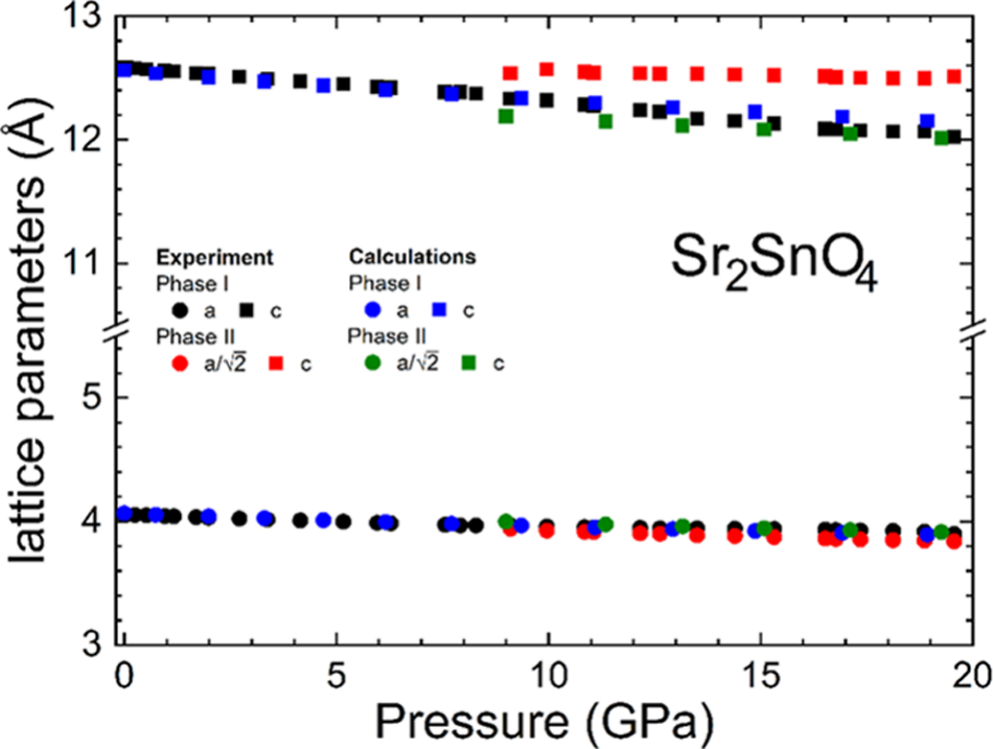
Pressure dependence of the unit-cell parameters of Sr_2_SnO_4_. Symbols are identified in the figure. Errors bars are smaller than symbols.

Figure 10 shows the pressure dependence of the unit-cell parameters for both the low- and high-pressure phases of Sr_2_SnO_4_. A table with these results is included in the Supporting Information (Table S3). The agreement between the experiment and calculation is good for both phases. In this compound, the compression is almost isotropic. For the low-pressure phase, we obtained linear compressibilities of κ_a_ = 2.21(9) 10^–3^ GPa^–1^ and κ_b_ = 2.07(9) 10^–3^ GPa^–1^.

**Figure 11:**
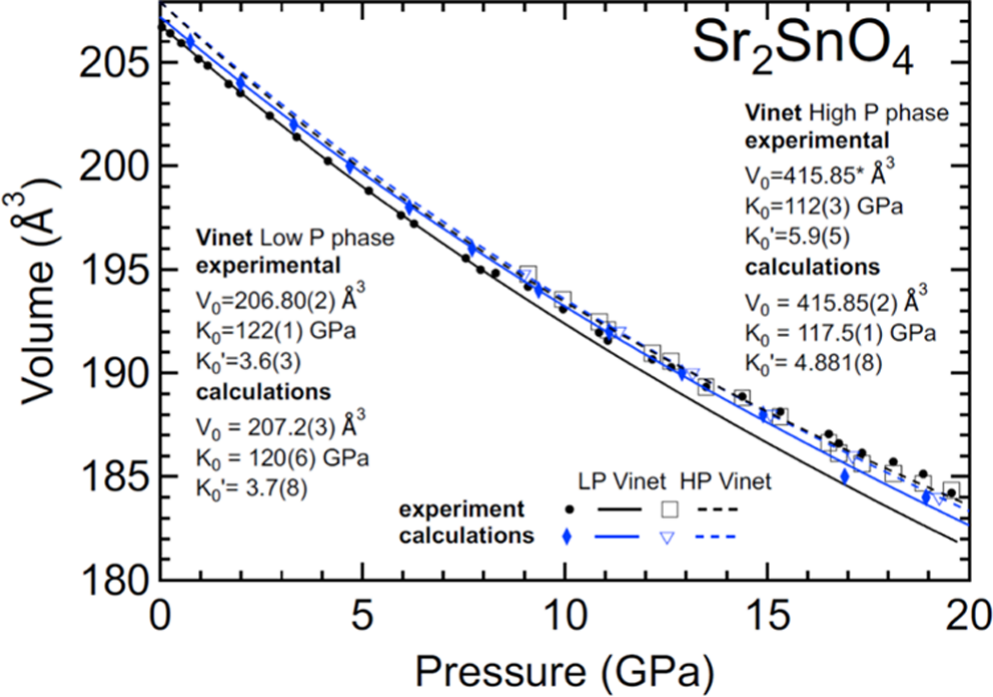
Pressure dependence of the unit-cell volume of Sr_2_SnO_4_. Symbols are results from experiments and calculations; lines represent the EOS fits. Details of each symbol/line are given inside the figure. For the HP phase, we plotted *V*/2 to facilitate comparison. Errors bars are smaller than symbols.

In Figure 11, we show the pressure dependence of the unit-cell volume. From these results, the parameters of the EOS (3rd-order Vinet) of the low-pressure phase are *V*_0_ = 206.80(5) Å^3^, *K*_0_ = 122(1) GPa, and *K*_0_′ = 3.6(3) according to experiments and *V*_0_ = 207.2(3) Å^3^, *K*_0_ = 120(6) GPa, and *K*_0_′ = 3.7(5) according to calculations. For the HP phase, we obtained *V*_0_ = 415.85(5) Å^3^, *K*_0_ = 112(3) GPa, and *K*_0_′ = 5.9(5) according to experiments and *V*_0_ = 415.85(2) Å^3^, *K*_0_ = 117.5(1) GPa, and *K*_0_′ = 4.881(8) according to calculations. Both phases exhibit a similar compressibility. In fact, if a second-order EOS is used, the bulk modulus of the low-pressure phase, 118(2) GPa, and high-pressure phase, 120(5) GPa, agree within uncertainties. The bulk modulus of both phases of Sr_2_SnO_4_ is similar to that of Ca_2_SnO_4_.

### 3.4 Mechanical Properties

In order to characterize the mechanical properties of the studied compounds, we calculated the elastic constants were calculated. The results are summarized in Table 1. They match the Born criteria of stability for the corresponding crystal system.^48^ From these constants, we have calculated the bulk modulus (*B*), shear modulus (*G*), Young’s modulus (*E*), and Poisson’s ratio (ν) using the Voigt and Reuss approximations. The results are summarized in Table 2. The average values obtained for the bulk modulus are comparable to those obtained from the EOS analysis of the experiments and the DFT calculations, confirming the conclusions of previous sections. In Zn_2_SnO_4_, we found that the Young’s modulus is 20% smaller than the bulk modulus, indicating that the resistance to tensile (or compressive) stress is lower than the resistance to volumetric compression. The opposite behavior was observed for Ca_2_SnO_4_ and Sr_2_SnO_4_. On the other hand, in all three compounds, the shear modulus was found to be considerably smaller than the bulk modulus, indicating that shear deformations are favored over volume contraction in M_2_SnO_4_ stannates, making them susceptible to nonhydrostatic stresses.^49^ The calculated Poisson’s ratios are between 0.26 and 0.36, which are typical values for solids. The Poisson’s ratio of Zn_2_SnO_4_ is comparable to that of copper, and the Poisson’s ratio of the other two compounds is comparable to that of steel. The value of the Poisson’s ratio for the three compounds indicates that the interatomic bonding forces are predominantly central and that ionic bonding is predominant over covalent bonding.

**Table 1:** Elastic Constants of Zn_2_SnO_4_, Ca_2_SnO_4_, and Sr_2_SnO_4_ Calculated by Using DFT

compound	C_11_ (GPa)	C_22_ (GPa)	C_33_ (GPa)	C_12_ (GPa)	C_13_ (GPa)	C_23_ (GPa)	C_44_ (GPa)	C_55_ (GPa)	C_66_ (GPa)
Zn_2_SnO_4_	200.3			150.2			74.0		
Ca_2_SnO_4_	250.1	170.6	251.9	85.1	59.4	88.4	78.4	48.5	70.3
Sr_2_SnO_4_	225.0		241.5	45.0	77.7		61.0		55.6

**Table 2:** Bulk Modulus (*B*), Shear Modulus (*G*), Young Modulus (*E*), and Poisson’ Ratio (ν) Calculated Using DFT for the Three Studied Compounds Using the Voigt and Reuss Approximations and Their Average

	Voigt	Reuss	average
Zn_2_SnO_4_			
*B*	166.9	166.9	166.9
*G*	54.4	41.5	47.9
*E*	147.2	115.0	131.1
ν	0.353	0.385	0.369
Ca_2_SnO_4_			
*B*	126.5	124.4	125.4
*G*	68.8	64.0	66.4
*E*	174.6	164.0	169.3
ν	0.270	0.280	0.275
Sr_2_SnO_4_			
*B*	121.4	120.1	120.8
*G*	68.2	66.1	67.2
*E*	172.4	167.6	170.0
ν	0.263	0.267	0.265

To conclude the discussion of our results, we will systematically investigate the bulk modulus of M_2_SnO_4_ stannates. For this purpose, in addition to the results presented in the previous sections, we will use results obtained from total energy calculations which have also been performed for Ba_2_SnO_4_ (isomorphic to Sr_2_SnO_4_),^7^ and the two polymorphs of Cd_2_SnO_4_ (which are isomorphic to Zn_2_SnO_4_ and Ca_2_SnO_4_^11^). The bulk moduli and average M–O and Sn–O bond distances are summarized in Table 3. To be consistent in the comparison, we use results from DFT calculations for all compounds studied in the work, all carried out under the same approximation. For the two polymorphs of Pb_2_SnO_4_, we have used the results reported by Sparh et al.^6^ The calculated EOS parameters for Ba_2_SnO_4_ are *V*_0_ = 115.1(5) Å^3^, *K*_0_ = 107(3) GPa, and *K*_0_′ = 5.2(3); for spinel-type Cd_2_SnO_4_, they are *V*_0_ = 780.0(5) Å^3^, *K*_0_ = 147(3) GPa, and *K*_0_′ = 4.9(3); and for spinel-type Cd_2_SnO_4_, they are *V*_0_ = 714.8(5) Å^3^, *K*_0_ = 141(3) GPa, and *K*_0_′ = 4.4(3).

**Table 3:** Bulk Modulus (*K*_0_) and Average M–O and Sn–O Bond Distances for Various Stannates. For the three compounds here studied, we present the experimental (EXP) and theoretical (DFT) bulk modulus; for Pb_2_SnO_4_, we used the result from ref (6); and for the rest of compounds, we include the DFT-calculated bulk modulus

compound (space group)	*K*_0_ (GPa)	average M–O bond distance (Å)	average Sn–O bond distance (Å)
Zn_2_SnO_4_ (*Fd*3̅*m*)	165(1) - DFT	2.0913(9)	1.9976(9)
	160(5) - EXP		
Cd_2_SnO_4_ (*Fd*3̅*m*)	147(3) - DFT	2.2440(15)	2.0759(15)
Cd_2_SnO_4_ (*Pbam*)	141(3) - DFT	2.3353(15)	2.0876(15)
Ca_2_SnO_4_ (*Pbam*)	125(1) - DFT	2.4391(15)	2.1009(15)
	125(2) - EXP		
Sr_2_SnO_4_ (*I*4/*mmm*)	120(6) - DFT	2.6665(17)	2.0391(15)
	122(1) - EXP		
Pb_2_SnO_4_ (*Pnam*)	117(6) – ref (6)	2.5505(17)	2.0616(15)
Ba_2_SnO_4_ (*I*4/*mmm*)	107(3) - DFT	2.7888(18)	2.0623(15)

**Figure 12:**
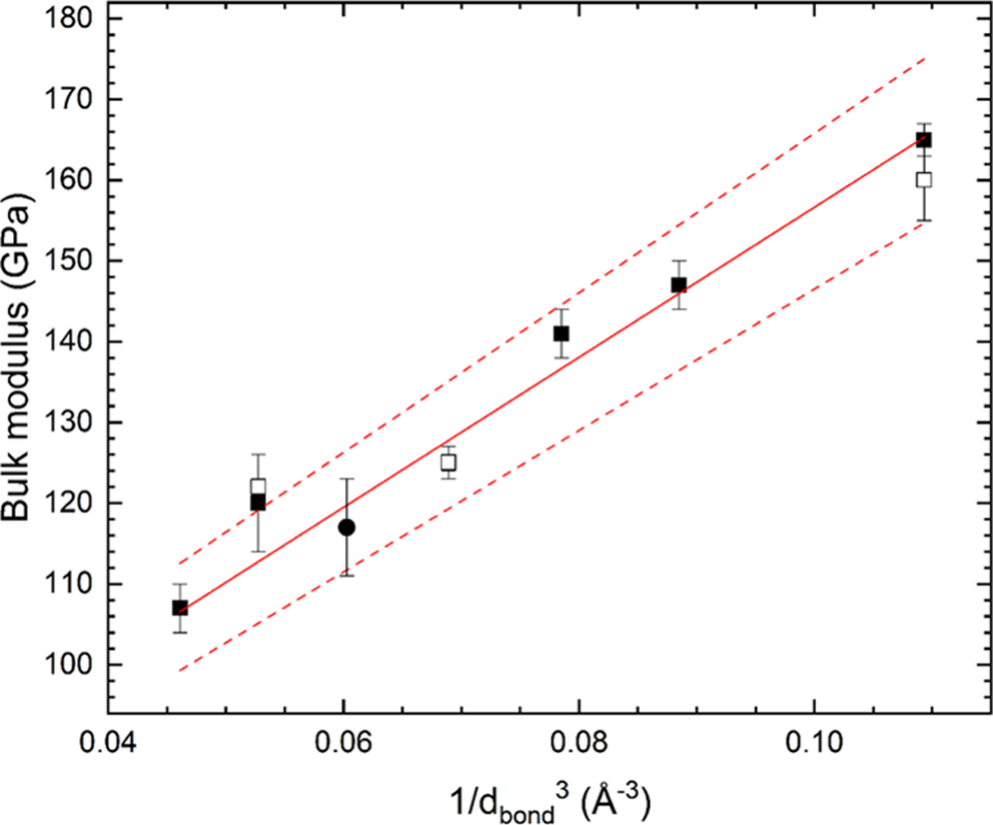
Bulk modulus versus 1/*d*^3^ representing the results from Table 3. Squares are from the present study. Solid (empty) squares are results from the present DFT calculations (experiments). The circle represents the bulk modulus of Pb_2_SnO_4_ obtained from ref (6). The dotted lines indicate the 99% confidence level band of the fit to the results. The crystal structure of each compound is indicated in Table 3.

It is known that in related compounds such as spinel oxides, the bulk modulus can be expressed in terms of polyhedral compressibility, which is usually inversely correlated with bond distances.^50^ In Table 3, it can be seen that in our study, the Sn–O bond distances are similar in all of the compounds and that there is no correlation between the bulk modulus and the average Sn–O bond distance. In contrast, there is an inverse correlation between average M–O bond distances and bulk moduli. Thus, to a first approximation, the bulk modulus is determined by the M–O distance. This situation is similar to that of other divalent metal ternary oxides, for instance, MTO_4_ oxides.^51^ For these compounds, a linear correlation has been found between the bulk modulus and 1/*d*^3^, where *d* is the average M–O distance. In Figure 12, we have plotted the bulk modulus versus 1/*d*^3^, for the compounds summarized in Table 3. In the figure, it can be clearly seen that the data follows a linear relationship, *K* = 63(4) GPa + 930(55) GPaÅ^3^ (1/*d*^3^), the *R*^2^ of the fit is 0.968.

The results presented in Figure 12 support the hypothesis that in M_2_SnO_4_ compounds, the bulk modulus is determined by the average M–O bands. The polymorph of Pb_2_SnO_4_ described by space group *Pbam* is an exception to this rule, as its bulk modulus is 36(2) GPa. Such a low bulk modulus is caused by the unusual coordination of the Pb atoms. In this polymorph, the Pb atoms are located at the apex of a trigonal pyramid bonded to three oxygen atoms in the base of the pyramid. The Pb atoms have an active lone electron pair pointing in the direction opposite to the base. The presence of the lone electron pair makes the structure highly compressible. This phenomenon has also been documented for other compounds with a lone electron pair cation, such as iodates,^52^ which have similar bulk moduli to the polymorph of Pb_2_SnO_4_ described by space group *Pbam*. The relationship we found can be used to estimate the bulk moduli of other M_2_SnO_4_ stannates. For instance, for spinel-type Mg_2_SnO_4_,^53^ Co_2_SnO_4_,^54^ Mn_2_SnO_4_,^55^ and Ni_2_SnO_4_,^56^ with an average M–O bond distance of 2.0740 Å, Co–O bond distance of 2.1085 Å, Mn–O bond distance of 2.1511 Å, and Ni–O bond distance of 2.0582 Å, bulk moduli are estimated to be 168(10), 163(10), 156(10), and 170(10) GPa, respectively.

## 4 Conclusions

Using high-pressure powder synchrotron XRD, we have shown that Zn_2_SnO_4_ and Ca_2_SnO_4_ do not undergo phase transitions up to 20 GPa. In the case of Zn_2_SnO_4_, our results solve the discrepancies observed in the literature for the phase transitions in this compound. In contrast, powder XRD provides evidence of a phase transition in Sr_2_SnO_4_ at 9.09 GPa. The transition is from the ambient-condition tetragonal polymorph (space group *I*4/*mmm*) to the low-temperature tetragonal polymorph (space group *P*4_2_/*ncm*). These conclusions are supported by density functional theory calculations. The room-temperature pressure–volume equation of state is reported for the three studied compounds and a systematic is established. We also found that compression is anisotropic in Ca_2_SnO_4_, nearly isotropic in Sr_2_SnO_4_, and isotropic in Zn_2_SnO_4_. We have also calculated elastic constants and moduli, which show that the studied compounds prefer shear compression to axial and volumetric compression. A systematic discussion of the high-pressure behavior of M_2_SnO_4_ stannates is presented, and predictions for Mg_2_SnO_4_, Co_2_SnO_4_, and Mn_2_SnO_4_ are also presented.

## Data Availability

The data that support the findings of this study are available from the corresponding author upon reasonable request.

## Supporting Information Available

Tables with unit-cell parameters versus pressure for different structures; figures with examples of profile matching of XRD patterns (PDF)
