# Supplementary material for: High-Pressure Behavior of Ca2SnO4, Sr2SnO4, and Zn2SnO4
Source: J Phys Chem C Nanomater Interfaces. 2024 Jan 12;128(3):1357–67. doi: 10.1021/acs.jpcc.3c06726 (PMC10823467; doi:10.1021/acs.jpcc.3c06726)
Supplement: Supplementary file 1 — jp3c06726_si_001.pdf [file jp3c06726_si_001.pdf]

Supplementary Information

High-Pressure Behavior of Ca_2SnO_4, Sr_2SnO_4, and Zn_2SnO_4

Simone Anzellini[1,*], Daniel Diaz-Anichtchenko[1], Josu Sanchez-Martin[1], Robin Turnbull[1], Silvana Radescu[2], Andres Mujica[2], Alfonso Muñoz[2], Sergio Ferrari[3,4], Laura Pampillo[3,4], Vitaliy Bilovol[3,5], Catalin Popescu[6], Daniel Errandonea[1]

[1]Departamento de Física Aplicada-ICMUV, MALTA Consolider Team, Universidad de Valencia, Edificio de Investigación, Carrer del Dr. Moliner 50, Burjassot, 46100 Valencia, Spain

[2]Departamento de Física, MALTA-Consolider Team, Instituto de Materiales y Nanotecnología, Universidad de La Laguna, San Cristóbal de La Laguna, E-38200 Tenerife, Spain

[3]Universidad de Buenos Aires, Facultad de Ingeniería, Departamento de Física, Laboratorio de Sólidos Amorfos, Av. Paseo Colón 850 (C1063ACV), Buenos Aires, Argentina

[4]CONICET - Universidad de Buenos Aires, Instituto de Tecnologías y Ciencias de la Ingeniería "Hilario Fernández Long" (INTECIN), Av. Paseo Colón 850 (C1063ACV), Buenos Aires, Argentina

[5]Academic Centre for Materials and Nanotechnology, AGH University of Science and Technology, Al. Mickiewicza 30, 30-059 Krakow, Poland

[6]CELLS-ALBA Synchrotron Light Facility, Cerdanyola, Barcelona 08290, Spain

a (Å)	V (Å³)	P (GPa)
8.656(2)	648.61(39)	0.18(2)
8.656(2)	648.55(39)	0.18(2)
8.654(2)	648.01(39)	0.28(2)
8.648(2)	646.70(39)	0.57(2)
8.648(2)	646.67(39)	0.58(2)
8.643(2)	645.60(39)	0.82(2)
8.637(2)	644.28(39)	1.15(2)
8.630(2)	642.66(39)	1.56(2)
8.621(2)	640.73(38)	2.01(2)
8.610(2)	638.17(38)	2.72(2)
8.595(2)	634.87(38)	3.65(2)
8.582(2)	632.13(38)	4.41(3)
8.563(2)	627.87(38)	5.64(3)
8.557(2)	626.49(38)	6.11(3)
8.545(2)	623.93(37)	6.84(3)
8.532(2)	621.03(37)	7.72(3)
8.519(2)	618.36(37)	8.56(3)
8.511(2)	616.48(37)	9.37(3)
8.506(2)	615.32(37)	9.84(3)
8.501(2)	614.34(37)	10.11(4)
8.497(2)	613.45(37)	10.64(4)
8.492(2)	612.33(37)	11.28(4)
8.486(2)	611.19(37)	11.95(4)
8.483(2)	610.37(37)	12.46(4)
8.478(2)	609.26(37)	12.99(4)
8.472(2)	608.13(36)	13.49(4)
8.468(2)	607.12(36)	14.00(4)
8.461(2)	605.73(36)	14.61(4)
8.454(2)	604.25(36)	15.66(5)
8.445(2)	602.22(36)	16.77(5)
8.437(2)	600.47(36)	17.28(5)
8.429(2)	598.87(36)	18.48(5)
8.421(2)	597.16(36)	19.63(5)
8.415(2)	595.90(36)	20.00(5)

Table S1: Lattice parameters of Zn_2SnO_4 as a function of pressure as determined from the Pawley refinement of its $Fd\overline{3}m$ structure.

a (Å)	b (Å)	c (Å)	V (Å^3)	P (GPa)
5.735(1)	9.664(2)	3.256(1)	180.48(10)	1.15(2)
5.734(1)	9.662(2)	3.256(1)	180.39(10)	1.23(2)
5.734(1)	9.662(2)	3.256(1)	180.38(10)	1.22(2)
5.731(1)	9.654(2)	3.254(1)	180.02(10)	1.49(2)
5.725(1)	9.640(2)	3.251(1)	179.39(10)	1.98(2)
5.717(1)	9.623(2)	3.247(1)	178.63(10)	2.55(2)
5.709(1)	9.604(2)	3.242(1)	177.76(10)	3.22(2)
5.702(1)	9.586(2)	3.238(1)	176.95(10)	3.88(2)
5.693(1)	9.566(2)	3.233(1)	176.10(10)	4.60(2)
5.685(1)	9.546(2)	3.228(1)	175.20(10)	5.34(3)
5.680(1)	9.533(2)	3.226(1)	174.66(10)	5.78(3)
5.671(1)	9.510(2)	3.220(1)	173.66(10)	6.67(3)
5.659(1)	9.480(2)	3.213(1)	172.39(10)	7.80(3)
5.647(1)	9.450(2)	3.207(1)	171.11(10)	8.98(3)
5.641(1)	9.436(2)	3.204(1)	170.55(10)	9.70(3)
5.635(1)	9.422(2)	3.201(1)	169.96(10)	10.51(4)
5.626(1)	9.396(2)	3.196(1)	168.94(10)	11.42(4)
5.618(1)	9.375(2)	3.192(1)	168.11(10)	11.97(4)
5.613(1)	9.361(2)	3.189(1)	167.54(10)	12.43(4)
5.597(1)	9.319(2)	3.181(1)	165.89(10)	13.64(4)
5.593(1)	9.307(2)	3.178(1)	165.44(10)	14.09(4)
5.586(1)	9.288(2)	3.174(1)	164.69(10)	14.73(4)
5.579(1)	9.267(2)	3.170(1)	163.90(10)	15.41(5)
5.570(1)	9.242(2)	3.165(1)	162.93(10)	16.09(5)
5.561(1)	9.217(2)	3.160(1)	161.96(10)	16.85(5)
5.547(1)	9.181(2)	3.152(1)	160.53(10)	17.91(5)

Table S2: Lattice parameters of Ca_2SnO_4 as a function of pressure as determined from the Pawley refinement of its *Pbam* structure.

a_{LP} (Å)	c_{LP} (Å)	V_{LP} (Å^3)	a_{HP} (Å)	c_{HP} (Å)	V_{HP} (Å^3)	P (GPa)
4.054(1)	12.581(3)	206.74(12)				0.00(2)
4.053(1)	12.581(3)	206.70(12)				0.04(2)
4.051(1)	12.577(3)	206.40(12)				0.25(2)
4.048(1)	12.569(3)	205.92(12)				0.52(2)
4.042(1)	12.556(3)	205.17(12)				0.95(2)
4.040(1)	12.551(3)	204.83(12)				1.17(2)
4.034(1)	12.536(3)	203.94(12)				1.69(2)
4.030(1)	12.529(3)	203.51(12)				1.98(2)
4.022(1)	12.510(3)	202.41(12)				2.71(2)
4.015(1)	12.494(3)	201.39(12)				3.37(2)
4.007(1)	12.473(3)	200.24(12)				4.15(2)
3.996(1)	12.449(3)	198.80(12)				5.16(3)
3.988(1)	12.426(3)	197.61(12)				5.96(3)
3.985(1)	12.419(3)	197.20(12)				6.28(3)
3.973(1)	12.387(3)	195.54(12)				7.56(3)
3.968(1)	12.385(3)	194.98(12)				7.92(3)
3.968(1)	12.371(3)	194.82(12)				8.29(3)
3.968(1)	12.330(3)	194.16(12)	5.574(1)	12.537(3)	194.77(12)	9.10(3)
3.960(1)	12.316(3)	193.09(12)	5.550(1)	12.568(3)	193.55(12)	9.96(3)
3.953(1)	12.283(3)	191.96(12)	5.539(1)	12.547(3)	192.46(12)	10.85(4)
3.950(1)	12.276(3)	191.57(12)	5.535(1)	12.539(3)	192.07(12)	11.06(4)
3.947(1)	12.239(3)	190.66(12)	5.519(1)	12.537(3)	190.96(12)	12.16(4)
3.945(1)	12.225(3)	190.28(12)	5.515(1)	12.532(3)	190.56(12)	12.63(4)
3.944(1)	12.171(3)	189.32(12)	5.497(1)	12.532(3)	189.33(12)	13.50(4)
3.942(1)	12.153(3)	188.85(12)	5.490(1)	12.527(3)	188.80(12)	14.39(4)
3.938(1)	12.129(3)	188.13(12)	5.478(1)	12.521(3)	187.88(12)	15.32(5)
3.934(1)	12.088(3)	187.06(12)	5.461(1)	12.514(3)	186.63(12)	16.52(5)
3.930(1)	12.082(3)	186.61(12)	5.456(1)	12.506(3)	186.13(12)	16.77(5)
3.926(1)	12.074(3)	186.14(12)	5.449(1)	12.501(3)	185.62(12)	17.35(5)
3.923(1)	12.065(3)	185.71(12)	5.444(1)	12.498(3)	185.16(12)	18.12(5)
3.917(1)	12.064(3)	185.13(12)	5.437(1)	12.496(3)	184.66(12)	18.86(5)
3.898(1)	12.121(3)	184.21(12)	5.428(1)	12.511(3)	184.33(12)	19.56(5)

Table S3: Lattice parameters of the LP (*I*4/*mmm*) and HP (*P*4$_2$/*ncm*) phases of Sr_2SnO_4 as a function of pressure as determined from the Pawley refinement. In order to compare the obtained volumes, the volume of the HP phase was divided by two.

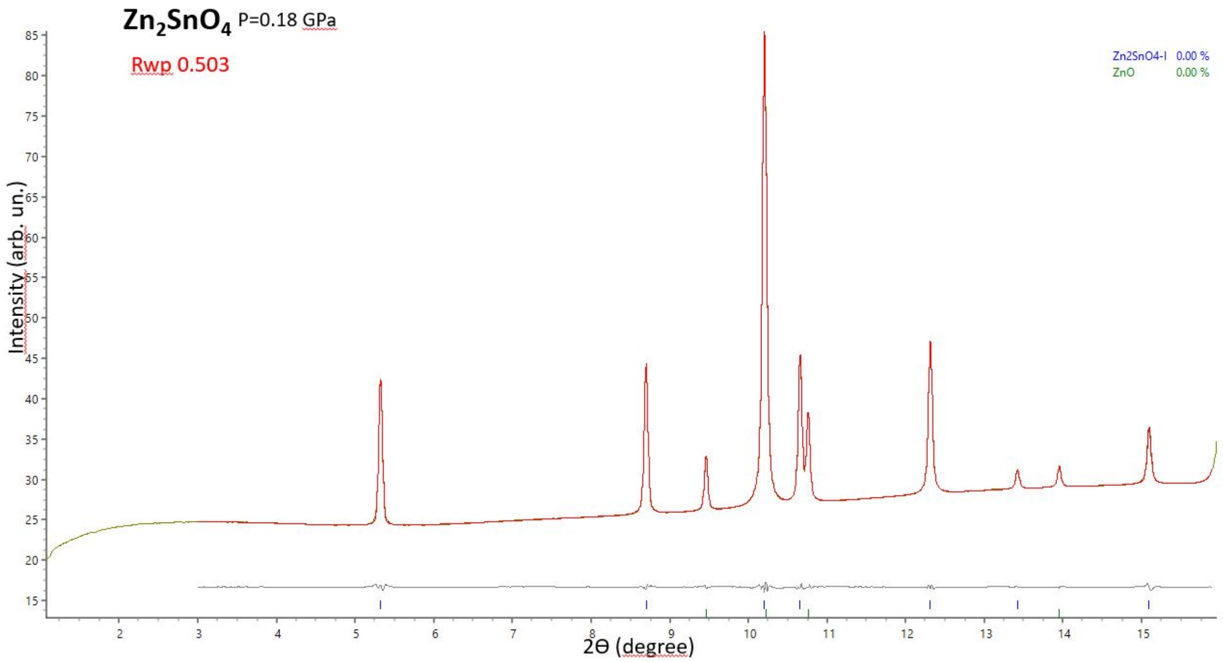

Figure S1: Pawley refinement of Zn_2SnO_4 at ambient P, where it is possible to see the good fitting of the data with the sample $Fd\overline{3}m$ phase.

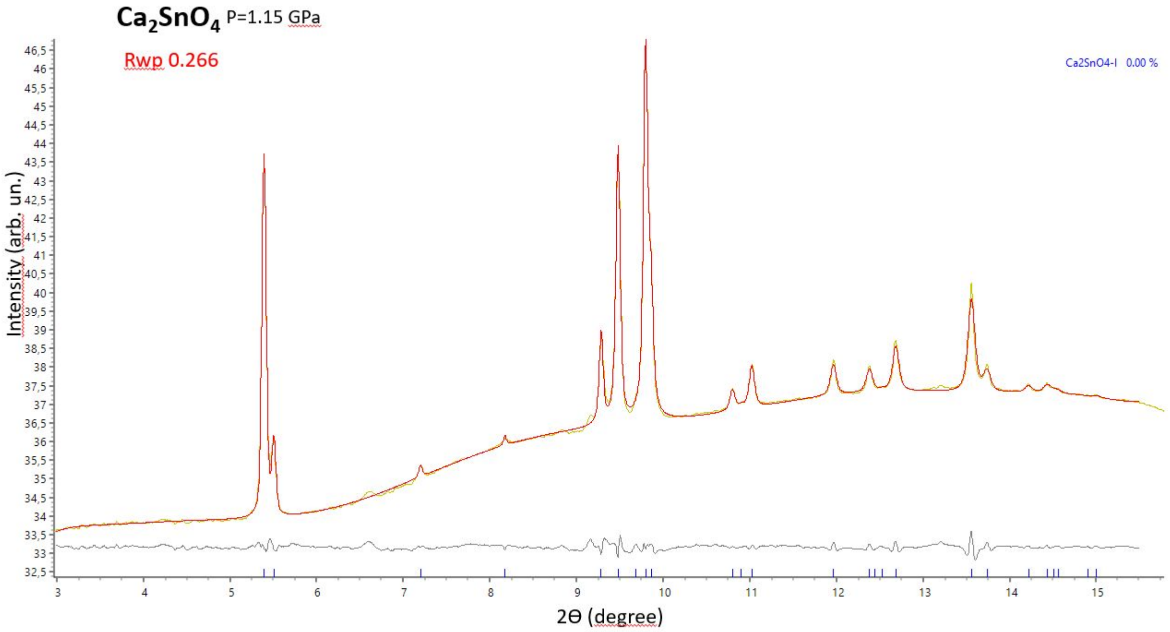

Figure S2: Pawley refinement of Ca_2SnO_4 at ambient P, where it is possible to see the good fitting of the data with the sample *Pbam* phase.

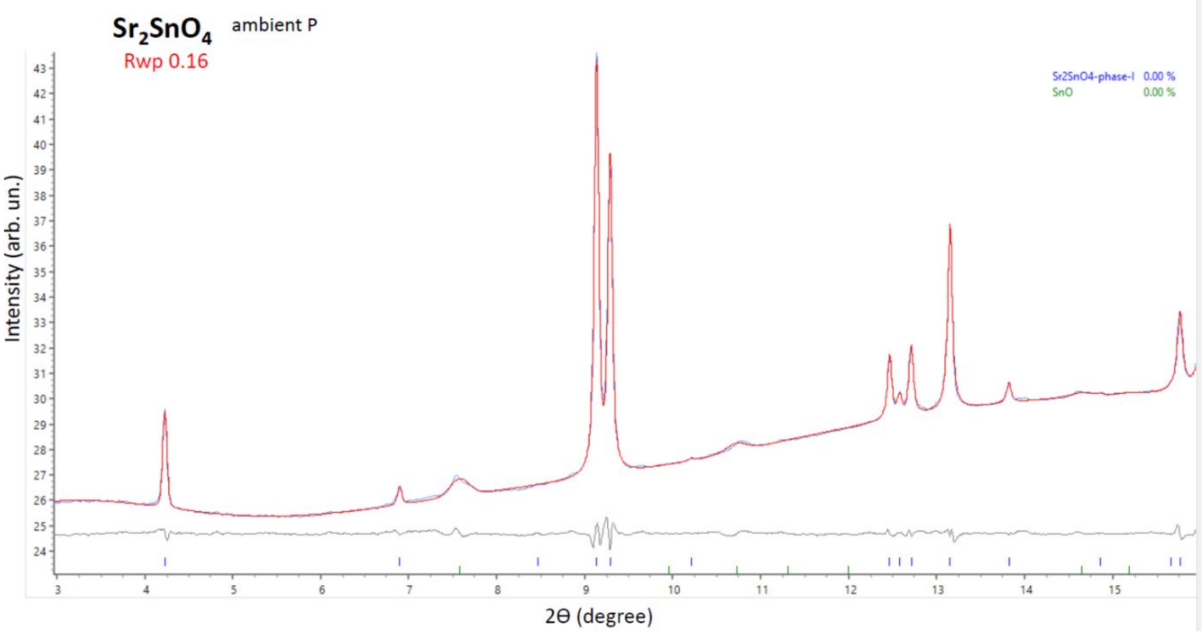

Figure S3: Pawley refinement of Sr_2SnO_4 at ambient P, where it is possible to see the good fitting of the data with the sample LP (*I*4/*mmm*) phase and the presence of the SnO_2 peaks.

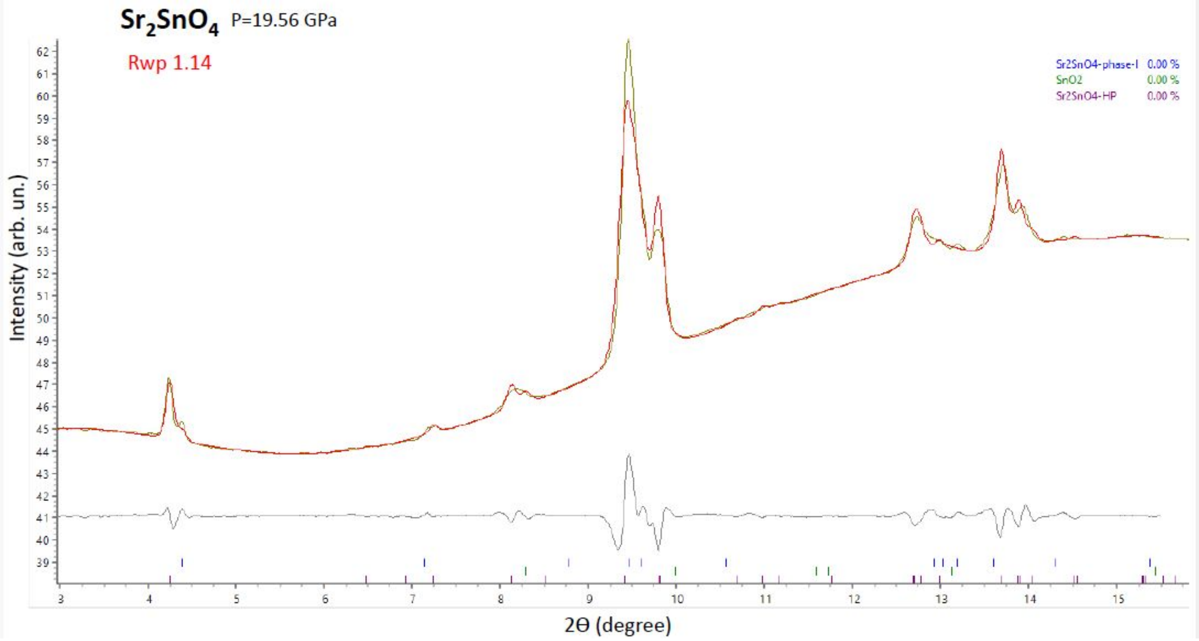

Figure S4: Pawley refinement of Sr_2SnO_4 at 19.56 GPa, where it is possible to see the good fitting of the data with the sample LP (*I*4/*mmm*) and HP (*P*4$_2$/*ncm*) phases and the presence of the SnO_2 peaks
